# Draft Genome Sequence of Paenibacillus polymyxa DSM 292, a Gram-Positive, Spore-Forming Soil Bacterium with High Biotechnological Potential

**DOI:** 10.1128/MRA.00071-20

**Published:** 2020-03-12

**Authors:** Simon Heinze, Ilias Lagkouvardos, Wolfgang Liebl, Wolfgang H. Schwarz, Petra Kornberger, Vladimir V. Zverlov

**Affiliations:** a Department of Microbiology, Technical University of Munich, Freising, Germany; b ZIEL Institute for Food and Health, Technical University of Munich, Freising, Germany; c Aspratis GmbH, Munich, Germany; d Institute of Molecular Genetics, Russian Academy of Science, Moscow, Russia; University of Arizona

## Abstract

Paenibacillus polymyxa DSM 292 was originally isolated from soil in 1947 due to its ability to produce antibiotics. The low proteolytic properties of strain DSM 292 warrant its examination as a host for heterologous protein production. Here, we report the draft genome sequence of DSM 292 as established by Illumina MiSeq paired-end sequencing.

## ANNOUNCEMENT

Paenibacillus polymyxa is the type species of the genus *Paenibacillus* (1), a genus of soil bacteria with a multitude of useful properties. Some examples include plant growth promotion (for example, through nitrogen fixation or production of phytohormones), production of antimicrobial compounds, such as polymyxins, and industrially relevant substances, like 2,3-butanediol or exopolysaccharides (2, 3).

In comparison to other strains of the same species, Paenibacillus polymyxa DSM 292 shows some distinguishing traits. *P. polymyxa* is readily amenable to genetic modification using the transmating procedure described by Heinze et al. (4). Most notably, strain DSM 292 did not form halos on milk agar plates, indicating a significantly lower exoprotease activity than strain DSM 365 or the type strain *P. polymyxa* DSM 36. This may lead to higher stability of heterologously expressed, secreted target proteins, making *P. polymyxa* DSM 292 an interesting candidate for production. This is a new application of this organism, meeting the increased demand for highly efficient expression systems to produce industrially relevant enzymes (5). *P. polymyxa* DSM 292 may be a promising alternative to the existing expression strains, which mainly belong to the genus *Bacillus* (5, 6).

As a basis for successfully working with *P. polymyxa* DSM 292, its genome was sequenced. The strain was obtained from DSMZ (Leibniz Institute DSMZ–German Collection of Microorganisms and Cell Cultures) and cultivated for 24 h at 37°C and 180 rpm in liquid LB medium (7). Chromosomal DNA was extracted from a clonal pure, stationary-phase culture, and 1 μg genomic DNA in total was used to prepare a DNA library with the TruSeq DNA PCR-free sample preparation kit (Illumina, Inc., San Diego, USA). A protocol for optimized DNA shearing and fragment size selection was applied to prepare the DNA library (8), which was sequenced using the Illumina MiSeq system in 150-bp paired-end mode according to the manufacturer’s instructions. The sequence data were assembled using SPAdes v3.6.1 with the BayesHammer tool for error correction and the MismatchCorrector for postassembly mismatch and indel corrections activated (9), resulting in 23 contigs with an average of 90.4-fold coverage. The assemblies were evaluated using QUAST v3.1 (10). Default parameters were used for all software unless otherwise specified. The combined length of the 23 contigs is 6,030,236 bp, with a G+C content of 45.5 mol% and an *N*_50_ value of 576,871 bp. Prokka v1.11 (11), with default parameters, was used for the prediction and annotation of open reading frames (ORFs). In this way, 5,365 coding sequences and 99 tRNAs were identified.

The genome sequence provided here will enable studies on *P. polymyxa* DSM 292 as a potential host for heterologous enzyme production, for example, by providing the basis for selection of genetic elements or targets for genome editing.

### Data availability.

This whole-genome shotgun project has been deposited in ENA under the accession number OXKC00000000. The version described in this paper is OXKC02000000. The GenBank assembly accession number is GCA_900406265.2. Raw sequencing reads are available at the Sequence Read Archive under accession number SRR10970105.

## References

[1] Ash C, Priest FG, Collins MD 1993 Molecular identification of rRNA group 3 bacilli (Ash, Farrow, Wallbanks and Collins) using a PCR probe test: proposal for the creation of a new genus *Paenibacillus*. Antonie Van Leeuwenhoek 64:253–260. doi:10.1007/bf00873085. 8085788

[2] Rütering M, Cress BF, Schilling M, Rühmann B, Koffas MAG, Sieber V, Schmid J 2017 Tailor-made exopolysaccharides—CRISPR-Cas9 mediated genome editing in *Paenibacillus polymyxa*. Synth Biol 2:ysx007. doi:10.1093/synbio/ysx007. PMC7445874 32995508

[3] Grady EN, MacDonald J, Liu L, Richman A, Yuan Z-C 2016 Current knowledge and perspectives of *Paenibacillus*: a review. Microb Cell Fact 15:203. doi:10.1186/s12934-016-0603-7. 27905924 PMC5134293

[4] Heinze S, Kornberger P, Grätz C, Schwarz WH, Zverlov VV, Liebl W 2018 Transmating: conjugative transfer of a new broad host range expression vector to various *Bacillus* species using a single protocol. BMC Microbiol 18:56. doi:10.1186/s12866-018-1198-4. 29884129 PMC5994095

[5] Küppers T, Steffen V, Hellmuth H, O'Connell T, Bongaerts J, Maurer K-H, Wiechert W 2014 Developing a new production host from a blueprint: *Bacillus pumilus* as an industrial enzyme producer. Microb Cell Fact 13:46. doi:10.1186/1475-2859-13-46. 24661794 PMC3987833

[6] Schallmey M, Singh A, Ward OP 2004 Developments in the use of *Bacillus* species for industrial production. Can J Microbiol 50:1–17. doi:10.1139/w03-076. 15052317

[7] Bertani G 1951 Studies on lysogenesis. I. The mode of phage liberation by lysogenic *Escherichia coli*. J Bacteriol 62:293–300. doi:10.1128/JB.62.3.293-300.1951. 14888646 PMC386127

[8] Huptas C, Scherer S, Wenning M 2016 Optimized Illumina PCR-free library preparation for bacterial whole genome sequencing and analysis of factors influencing de novo assembly. BMC Res Notes 9:269. doi:10.1186/s13104-016-2072-9. 27176120 PMC4864918

[9] Bankevich A, Nurk S, Antipov D, Gurevich AA, Dvorkin M, Kulikov AS, Lesin VM, Nikolenko SI, Pham S, Prjibelski AD, Pyshkin AV, Sirotkin AV, Vyahhi N, Tesler G, Alekseyev MA, Pevzner PA 2012 SPAdes: a new genome assembly algorithm and its applications to single-cell sequencing. J Comput Biol 19:455–477. doi:10.1089/cmb.2012.0021. 22506599 PMC3342519

[10] Gurevich A, Saveliev V, Vyahhi N, Tesler G 2013 QUAST: quality assessment tool for genome assemblies. Bioinformatics 29:1072–1075. doi:10.1093/bioinformatics/btt086. 23422339 PMC3624806

[11] Seemann T 2014 Prokka: rapid prokaryotic genome annotation. Bioinformatics 30:2068–2069. doi:10.1093/bioinformatics/btu153. 24642063

